# The Problem of Abnormal Body Weight and Dyslipidemia as Risk Factors for Cardiovascular Diseases in Children and Adolescents with Type 1 Diabetes

**DOI:** 10.1155/2021/5555149

**Published:** 2021-08-02

**Authors:** Katarzyna Noras, Ewa Rusak, Przemysława Jarosz-Chobot

**Affiliations:** ^1^Department of Children's Diabetology, Upper Silesian Child Health Centre, Katowice, Poland; ^2^Department of Children's Diabetology, Medical University of Silesia, Katowice, Poland

## Abstract

Diabetes is a disease that affects many people around the world. Its complications are the cause of cardiovascular diseases (CVD) and increased mortality. That is why the search for predictive biomarkers is so important. The aim of the study was to show the prevalence of the problem and risk factors in children and adolescents with type 1 diabetes. These patients are often overweight and obese, and the percentage of lipid disorders is particularly high. The discussed markers of CVD risk in type 1 diabetes include apolipoproteins (apo-B and apo-C3), modified forms of LDL, and the role of high-density lipoprotein (HDL). Recently, a new look at the vasoprotective effect of HDL has appeared, which due to its dysfunctional form in type 1 diabetes may not protect against cardiovascular risk. The HDL proteome in type 1 diabetes has an altered protein composition compared to the healthy population. Another direction of research is determining the importance of trace elements (mainly Mg) in the development of diabetes complications.

## 1. Introduction

Early prevention of chronic complications of diabetes is of great significance and continues to challenge many professionals. The duration and severity of hyperglycemia are the main factors causing tissue damage and contribute to the development of complications including both microangiopathies (retinal, glomerular, or nerve dysfunction) and macrovascular complications (earlier occurrence of more advanced atherosclerotic changes). Premature atherosclerosis of the arteries, which is the cause of cardiovascular diseases (CVD), is considered to be the main risk factor of almost three times higher mortality in type 1 diabetes [1, 2]. Moreover, advanced complications of diabetes mellitus, such as severe visual impairment, renal replacement therapy, consequences of neuropathy, or sexual dysfunction, restrict patients socially and deteriorate their quality of life. In view of the current increase in the prevalence of type 1 diabetes in children and adolescents, identification of predictive biomarkers and regular screening is very important. The aim of this article is to present the current knowledge about known risk factors for cardiovascular diseases as well as to present the directions of searching for new markers.

## 2. The Scale of Overweight and Obesity and Dyslipidemia as Risk Factors for Complications in Diabetes

Commonly known risk factors for cardiovascular diseases are excessive body weight and the atherogenic lipid profile correlating with it. The problem of overweight and obesity, which also includes children and adolescents with type 1 diabetes, is related to the globally observed change in eating habits. The prevalence of overweight in these patients, depending on the adopted criterion of overweight and obesity (i.e., BMI ≥ 85 pc or ≥95 pc, BMI ≥ 1SDS or ≥2SDS; as well as being an index of central obesity, waist circumference > 90c by gender and age; or WHtR > 0.5 (waist to height ratio) being the quotient of waist circumference and height) varies between 16% and 37%. Overweight is more often observed than obesity. The large scale of this problem is indicated by the fact that the abovementioned data is concerned with children with type 1 diabetes from different regions of the world [3–7].

Particularly worrying is the results of a 4-year retrospective study by Jones et al. of 565 children with type 1 diabetes mellitus from Bangladesh. The presence of risk factors for CVD, mainly lipid disorders, was estimated at over 60%. Abnormal levels of total cholesterol (TC), LDL-C, and HDL-C were found in 63.5%, 34.2%, and 22.0%, respectively [7]. An unmodified risk factor, which was associated with both a higher incidence of overweight and an impaired lipid profile, was female gender [3, 4, 8].

Independent risk factors for cardiovascular diseases are stimulants, especially nicotinism. According to the Italian observation of 228 adolescents with DMT1, this problem may concern even 1/4 of young patients, among whom cigarette smoking declared 10%, alcohol use 10%, and both alcohol and cigarette use 6% [9].

An interesting analysis was undertaken by Brazilian researchers who adopted HTGW (hypertriglyceridemic waist phenotype) as a marker of cardiovascular diseases, being an alternative to the metabolic syndrome. This index is defined as the cooccurrence of increased waist circumference (>90 percentile for gender and age) and increased triglyceride levels (TG) according to the NCEP (National Cholesterol Education Program) criterion, i.e., ≥75 mg/dl in children and ≥90 mg/dl in adolescents. The incidence of HTGW in children and adolescents with type 1 diabetes was 23.5%. It was associated with overweight and elevated levels of total cholesterol (TC), LDL-C, and VLDL limit values [8].

The great importance of achieving and/or maintaining proper body weight in the treatment of diabetes is emphasized by the population prospective Diabetes in Youth Study conducted over a decade (2002-2012), among young people with diabetes (3954 with DMT1 vs. 706 with DMT2). In this study, modified criteria of metabolic syndrome were used as cardiovascular diseases (CVD) risk factor: waist circumference > 90 c, TG concentration ≥ 110 mg/dl, HDL-C concentration ≤ 40 mg/dl, and HA ≥ 90 c. At the time of inclusion, the average duration of diabetes was 0.8 years for DMT1 and 1.0 for DMT2. The prevalence of ≥2 CVD risk factors was lower in patients with type 1 diabetes mellitus. Nevertheless, the analysis showed that, with the increase of abnormal waist circumference, their number increases, which was observed especially in children with type 2 diabetes [10].

The relationship between excessive body weight and insufficient diabetes control (HbA1c > 7.5%) and elevated levels of total cholesterol (TC), LDL-C, and triglycerides (TG) was also demonstrated by Polish observation of adolescents with type 1 diabetes. However, dyslipidemia was more frequently observed in adolescents with well-controlled diabetes, but obese or overweight, compared to the group with unsatisfactory diabetes control but normal body weight [3].

An extensive analysis of lipid profile in 14,290 children and adolescents with type 1 diabetes (up to the age of 18) based on the SWEET International Registry of Diabetes also confirmed the established relationship between dyslipidemia (increase in LDL or non-HDL cholesterol) and weight gain, duration of diabetes, or degree of glycemic control (HbA1c). The duration of diabetes mellitus and HbA1c were grouped as follows: <5 years, 5-10 years, and >10 years and HbA1c < 7.5%, 7.5-9%, and >9%. The assessment of the influence of the diabetes treatment model on the metabolism spoke in favor of pump therapy compared to intensive insulin therapy with multiple daily injections and was associated with better lipid profile control [11].

The prospective SEARCH study of 1800 young patients with type 1 diabetes showed that the frequency of dyslipidemia (higher non-HDL cholesterol and/or lower HDL cholesterol) was estimated at 29%. Dyslipidemia with longer disease duration, poor glycemic control (HbA1c ≥ 9%), obesity, and elevated blood pressure correlated with recognized cardiovascular risk factors—arterial stiffness index (SI) and augmentation index (AIx). The evaluation of arterial stiffness (measurement of the speed of the arterial wave) and the augmentation index, which is a measure of the size of the reflected wave, characterizing the degree of vessel elasticity, was performed by tonometry using Sphygmocor CPVH. The incidence of increased reflections of the waves/arterial stiffness index was higher in diabetic patients [6].

## 3. Pathomechanism of Dyslipidemia and Search for New Biomarkers of CVD Risk in Diabetes Mellitus with Particular Emphasis on the Role of Dysfunctional HDL

The atherogenic lipid profile, well documented especially in type 2 diabetes, is characterized by high levels of triglycerides (TG), both fasting and after meal, reduced levels of HDL cholesterol (HDL-C) and normal or slightly elevated levels of LDL cholesterol (LDL-C). In this profile, attention is drawn to the changed composition of lipoproteins composed of a lipid core (rich in TG and cholesterol esters) and a polar shell (containing phospholipids, apolipoproteins, and free cholesterol). It is associated with the presence of dysfunctional HDL and small, dense LDL (sdLDL), the affinity of which for the LDL receptors of liver cells is reduced due to greater susceptibility to oxidation or nonenzymatic glycation. Consequently, their serum presence is prolonged and binding to the arterial wall increases [1, 12]. Accumulation of lipoproteins containing, e.g., apo-B, apo-E, and apo-C3 apolipoproteins, in the arterial wall accelerates the accumulation of macrophages, the formation of foam cells, and the process of atherogenesis [13] Thus, apolipoprotein-B (apo-B), a carrier protein of low-density lipoproteins (LDL) and very low-density lipoproteins (VLDL), seems to be a marker of cardiovascular disease risk, but its practical usefulness is limited mainly due to the cost of determination [12].

Advanced search for markers of cardiovascular disease risk in type 1 diabetes, extended by the analysis of the composition of modified LDL forms present in the circulating immune complexes (LDL-IC), was carried out among the participants of the prestigious, multicenter DCCT/EDIC study. A correlation has been demonstrated between the level of ox-LDL (oxidized LDL), MDA-LDL (malondialdehyde-modified LDL), and AGE-LDL (glycosylation-modified LDL) in circulating immune complexes (ICs) with serious cardiovascular diseases and events and myocardial infarction [14].

Experiments in mouse models with type 1 diabetes show that the relative insulin deficiency (which occurs in suboptimally controlled diabetes) increases the serum levels of apolipoprotein C3 and is associated with accelerated atherosclerosis. As shown in the study, the use of apoC3 antisense oligonucleotides (ASO) removed increased hepatic apoC3 expression which prevented accelerated atherogenesis [13].

There are also reports suggesting that high-density lipoprotein (HDL), commonly regarded as a vasoprotective particle, does not provide protection against cardiovascular risk in type 1 diabetes due to its dysfunctional form [1, 15]. An example is a paper published by Chiesa et al. evaluating the functional properties of HDL by means of endothelial nitric oxide (NO) bioavailability, superoxide (SO) production, and serum paraoxonase activity (PON-1) with the evaluation of endothelial function in a group of teenagers with type 1 diabetes (mean age: 14.6 years, mean HbA1c: 8.3%). In order to assess the severity of inflammation, a risk assessment was made using five proinflammatory markers (EGF: epidermal growth factor; GRO: oncogene regulated by chemokine growth; PDGF-AA and PDGF-BB: platelet growth factors AA and BB; and cCD40L: soluble CD-40 ligand). Increased inflammation index and HDL dysfunction have been demonstrated in patients with early renal dysfunction as assessed by the ACR (albumin/creatinine ratio). In contrast, impaired endothelial function has been reported in patients with high inflammation index and high HDL cholesterol (HDL-C) levels. This fact suggests that a high HDL-C level may be an unfavorable predictor of endothelial function, especially in the presence of chronic inflammation or renal dysfunction [16].

An attempt to assess HDL functionality was also undertaken in a 5-year follow-up of a group of 293 teenagers with type 1 diabetes (mean age: 13.7 years, mean HbA1C: 8.4%) compared to the control group. The HDL function was quantitatively determined by HAE-HDL-apoA-I exchange. Apolipoprotein A-I (apoA-I), which is the main component of the HDL protein, determines its shape and size, takes part in the activation of the lecithin-cholesterol acyl transferase (LCAT) enzyme, and plays an important role in removing cholesterol from peripheral cells by delivering the resulting cholesterol esters to the liver. This protein is considered to be the main component responsible for the antiatherosclerotic effect of HDL. In the above study, the HAE-apoA-I ratio was quantitatively determined, showing that this ratio was lower in diabetic patients and inversely correlated with the HbA1c level. The differences in HDL function appeared soon after the disease onset and persisted over time [17].

The issue of dysfunctional HDL particles was also analyzed in a study by Gourgari et al., in which a broad assessment of HDL protein composition was made in a small group of 26 patients with type 1 diabetes (mean age 16.9 years; mean HbA1c 8.9%; mean HDL 59.7 mg/dl). Comparative assessment of HDL proteomes in adolescents with diabetes compared to a healthy control group was performed. The analysis was carried out using mass spectrometry by quantitative determination of 78 proteins bound with HDL. The altered protein composition of HDL in adolescents with diabetes mellitus compared to the control group was confirmed, despite similar HDL concentration as, for example, significantly increased amounts of complement factor H2 protein (FHR2: factor H-related protein 2), regardless of glycemia. A further analysis of the patients showed that the level of glycemia had an influence on the level of some proteins, i.e., the alpha-1-beta glycoprotein or the alpha-4 trypsin inhibitor [18].

The literature also raises an interesting issue of the relationship between lowered magnesium levels and poor glycemic control and the presence of dyslipidemia in patients with type 1 diabetes. There are reports showing the relationship of hypomagnesemia with an increased risk of atherosclerosis [19] and increased levels of total cholesterol (TC), LDL, and triglyceride fraction cholesterol (TG) and lowered HDL levels [20]. An example of this is the publication by Shahbah evaluating the effect of oral Mg supplementation on glycemic control and lipid profile in children with type 1 diabetes mellitus and coexisting hypomagnesemia (Mg < 1.7 mg/dl). Already after 3 months of oral magnesium oxide supplementation at a dose of 300 mg/day, a significant improvement in glycemic control was observed (HbA1c 10.11% vs. 7.88%) along with a decrease in serum total cholesterol, LDL, and TG concentrations, as opposed to HDL, which increased significantly [21].

## 4. Summary

Despite pharmacological and technological progress, diabetes still remains one of the most serious and common health problems. Maintaining proper body weight in patients with this condition is very important to reduce the risk of cardiovascular disease. In order to be able to take even more targeted and effective action in the future to prevent the chronic complications of diabetes early, further research is needed to better identify predictive biomarkers. This is especially true for lipid disorders, where both quantitative changes and changes in the function of individual lipoprotein fractions are important. Further research will help to better understand the complex pathomechanism of dyslipidemia and its influence on glycemic control. Perhaps an appropriate model of treatment for diabetes and the associated better glycemic control will contribute to reversing HDL dysfunctional abnormalities which could potentially reduce the risk of vascular complications. Children with type 1 diabetes could particularly benefit as early preventive measures will improve their life expectancy and quality of life.
